# Using Design Thinking to Develop a Tool for Strengthening Nurses’ Cultural Competence: Lessons Learned

**DOI:** 10.1177/10436596251370604

**Published:** 2025-10-05

**Authors:** G. Duran-Kiraç, R. Uittenbroek, H. van Hout, M. I. Broese van Groenou

**Affiliations:** 1 Windesheim University of Applied Sciences, Zwolle, The Netherlands; 2 Vrije Universiteit, Amsterdam, The Netherlands; 3 Amsterdam University Medical Centers, Amsterdam, The Netherlands

**Keywords:** design method(s), design thinking, cultural competence, migrants, nurses

## Abstract

**Introduction:**

Nurses working in dementia care lack cultural competence to improve access to health care for ethnic minority persons. This study aimed to develop a tool to strengthen nurses’ cultural competence, using a Design Thinking approach in the Netherlands.

**Method:**

This qualitative participatory study followed five Design Thinking phases: empathize, define, ideate, prototype, and test. Data were collected during co-creation sessions with a Design Team consisting of nurses, an informal caregiver, researchers and students.

**Results:**

The Design Team (*n* = 7) formulated the problem statement: “It is difficult to map all involved in the care for the person with dementia, and to be able to ask right questions.” In response, the Good Conversation Method was developed, including a genogram to visualize the care network and conversation cards to explore cultural and religious identity.

**Discussion:**

Design Thinking lessons included the importance of including all stakeholders, managing differing perspectives, and allowing time for iterative development.

## Introduction

Ethnic minority (EM) people are at higher risk of developing dementia, yet they use health care services, homecare, and nursing homes less frequently than persons of ethnic majority (Bindraban et al., 2008; Duran-Kiraç et al., 2022, 2023; Parlevliet et al., 2016). Studies reported several barriers experienced by persons with dementia (PwD) with an EM background suggesting a need for culturally appropriate health care (Blix & Munkejord, 2022; Duran-Kiraç et al., 2022). This requires more understanding of how health care providers can contribute to timely diagnosis of dementia among EM groups, which can contribute to better access to culturally appropriate health care (Kenning et al., 2017), but a diagnosis on its own is insufficient for subsequent culturally appropriate use of services and home care. Nurses’ awareness of patients’ attitudes, readiness to seek help, and limited knowledge about health care services and home care is important (Duran-Kiraç et al., 2022), as it may lower barriers to accessing health care and support more responsive, needs-based professional care(Gove et al., 2021).

Strengthening cultural competences for health care professionals is needed, as cultural and religious values influence care needs, communication styles and preferences, and help-seeking behavior (Berdai Chaouni & De Donder, 2019; Clark et al., 2018; Duran-Kiraç et al., 2023). However, professionals often feel incompetent in conversation with persons with EM backgrounds. It is not clearly outlined how professionals can be best supported. There are several tools available that can assess cultural competence and its underlying dimensions (Javier & Belgrave, 2015; Sutharson et al., 2025), but these do not assist in acquiring and developing cultural competence. This is better captured in several conversation-support tools, such as the Cultural Formulation Interview. This is a tool used by mental health professionals to gather information about someone’s cultural background and how culture influences the mental health of patients (Lewis-Fernández et al., 2020). Yet, this interview seems less suitable for PwD and their wishes or barriers in using health services and home care. Nurses often engage with the relatives of PwD, which is also lacking in this Cultural Formulation Interview. At present, there is a need for a tool that strengthens the cultural competence of nurses working with PwD and an EM background and their informal caregivers.

Design Thinking might be a suitable approach to co-create a tool to improve cultural competence with stakeholders. It offers the opportunity to find a solution to a problem experienced in practice, with active involvement from stakeholders through co-creation (Roddy & Polfuss, 2020). Design Thinking can be especially useful in designing interventions for underserved populations whose needs may be overlooked by other approaches (Altman et al., 2018). Although Design Thinking has a history in engineering, architecture, and education, it recently also emerged in health care professions (Wolcott et al., 2021). For example, Aaronson et al. (2020) found in their study that Design Thinking offered a good method for process improvement regarding the patient-provider communication in the emergency department. Roddy and Polfuss (2020) indicate that combining Design Thinking with the expertise of health care professionals can provide an opportunity for them to influence product development. In addition, health care professionals’ involvement in the design process allows them to apply their expertise and influence products and services that support their work (Roddy & Polfuss, 2020). It is not yet known whether and in what way we can use design thinking to develop a tool to improve access to care for PwD and an EM background. The aim of this study was to develop a tool that can strengthen cultural competence of nurses who work with EM people through design thinking and to reflect on the development process.

## Method

The development of the tool was part of a larger project on health care accessibility of EM PwD that was executed between 2019 and 2025 in three regions in the Netherlands. This study builds on information collected in an earlier phase of the project. The aim of this study is to describe how a tool can be developed for practical use based on research principles. This study focused on the development process itself, rather than on evaluating the tool’s effectiveness. We followed a qualitative approach according to participative action research and applied the Design Thinking method, adopted from Stanford University’s D-school, between September 2021 and July 2022. We used the COREQ (Tong et al., 2007) checklist to assess our study’s quality (Online Supplemental Material), which showed that the quality is sufficiently high.

### Design Thinking

Design Thinking is a five-phase process that aims to explore, define, and solve problems. It has a user-centered focus and supports understanding the user experience and their challenges. Design Thinking consists of five phases: empathize, define, ideate, prototype, and test. In the empathize phase, stakeholders need to understand the problem and put themselves in the shoes of those affected. The define phase focuses on defining a problem statement. During the ideate phase, participants should feel free to express what they consider important without being hindered by potential obstacles. In the prototype phase, a prototype is developed, and in the test phase, the prototype is tested. Design Thinking is an iterative process, meaning that you can move back and forth between the different phases until the optimal solution is achieved.

### The Design Thinking Team

The first step of Design Thinking is creating a Design Team (DT) consisting of stakeholders. For the participation of PwD, we first contacted informal caregivers. Conversations revealed that the PwD were unable to participate. Therefore, we decided to invite close family members. Our DT consisted of a nurse who is an expert in dementia case management, two practice nurses in geriatric care, an informal caregiver with an EM background, and two nursing students. We included students to ensure a diverse range of experiences within the DT, involving stakeholders with varying levels of experience in working with PwD with an EM background. The DT was complemented by two researchers, the first and second authors. The first author is part of the research group Living Well with Dementia within the department of Health and Well-being and has experience in conducting research on health care accessibility for EM people. The second author is head of the Health and Social Care research department and has experience in conducting research on the experiences of case managers within people-centered care programs. The nurses who participated in the DT work for health care organizations that were part of the overarching project. They shared experiences on access to health care for EM people. Inclusion criteria for these nurses were that they worked with EM people. The informal caregiver who participated was recruited through the first author’s network and was also part of the overarching project. Inclusion criteria for the informal caregiver were having an EM background and currently providing care for a PwD.

#### Process Facilitator

An independent process facilitator (S.K.) led the DT through the Design Thinking process. Her role was to ensure that the Design Thinking sessions ran in a structured manner, to promote equal participation among all stakeholders within the DT, to mediate and resolve any conflicts of those arose. Her presence allowed researchers to fully engage as members of the DT. The process facilitator prepared each session by defining the goals for each phase of Design Thinking and designing concrete exercises or discussions to guide the DT though the process. Decisions during the Design Thinking process were made collectively, with a strong emphasis on ensuring that all voices were heard and considered. The goal was to reach consensus among all participants. The process facilitator made efforts to balance contributions so that no one dominated.

### Data Collection

Data collection followed the five phases of Design Thinking with the DT for a total of eight meetings. For this purpose, writable and reusable materials were used to follow the various steps of the Design Thinking process. All eight meetings with the DT were also audio-recorded, and the first and second authors took notes. Before each meeting, participants of the DT were verbally asked for permission to record the audio.

#### Emphasize

First, the DT empathized with nurses and the PwD with an EM background. To support the DT in this phase, they were given information and insights about the outcomes of previous studies within the larger project on cultural competence of nurses and what experiences of PwD with an EM background and their informal caregivers were concerning access to (appropriate) health care (Duran-Kiraç et al., 2022; Duran-Kiraç et al., 2023). The researcher presented barriers in health care access experienced by nurses and informal caregivers (see Table 1), mainly based on the theoretical framework of Levesque et al. (2013). For each insight into the experienced obstacles, examples from practice were provided, sourced from the interviews. The DT then split into two groups to discuss the most pressing obstacles to health care access, aiming to identify and assess whether these challenges were also recognized by all DT members.

**Table 1.** Barriers in Health care Experienced by Health care Professionals (Supply) and Informal Caregivers (Demand), Supplemented With Experiences From the DT Presented in Italics.

Approachability (supply)	Acceptability (supply)	Appropriateness (supply)
Professionals perceive communication as challenging	Professionals experience feelings of uncertainty because of cultural differences	Professionals have a need for cultural skills
Professionals experience a language barrier	Professionals have assumptions about ethnic minorities	Professionals have a need for more cultural knowledge
Professionals have insufficient knowledge of available resources to engage with EM groups	*Professionals feel unsure about where their tasks end*	Professionals have a need for skills to acquire cultural skills
Difference between contact person and decision-maker within the family of the client is unclear for the professionals		Professionals have a for being able to ask the right questions
*Professionals experience building and maintaining a relationship as difficult*		Professionals having fear of addressing (cultural) sensitive topics
*Professionals experience discrepancy in understanding dementia between informal caregiver and healthcare professional*		*Family members do not appear to be on the same page; no action can be taken*
Ability to perceive a need for healthcare (demand)	Ability to search healthcare (demand)	Ability to engage in healthcare (demand)
Informal caregivers and family members experience a lack of knowledge about dementia before diagnosis	Personal and social values of the informal caregiver can influence choices regarding whether or not to seek professional care	Informal caregivers experience a lack of person-centered care
Different health beliefs between professionals and persons with an ethnic minority background	Informal caregivers have a lack of knowledge about available care options and what to ask professionals	External (professional) help can cause shame and diminish pride within the informal caregiver
*Taking care of the older adult is common within some cultures, making signs of dementia less likely to be problematized*	*If symptoms are perceived as normal no help will be sought. If symptoms are not experienced as a disease, no solution will be sought*	Informal caregivers have a preference for staying involved in caregiving tasks
*Lack of health literacy within ethnic groups*	*Language barrier makes seeking healthcare more difficult*	*Informal caregiver feels caught between family, professional healthcare and the community*
	*The decision-maker within a family determines the acceptance of professional healthcare*	*Uncertainty and lack of knowledge about what to expect from professional healthcare*

#### Define

In this phase, the DT defined a problem statement. With the input from scientific insights (Duran-Kiraç et al., 2022; Duran-Kiraç et al., 2023) and bringing their own experiences, the DT discussed the most urgent problem(s) to address. Topics included difficulties in identifying all informal caregivers, communication barriers, and lack of cultural sensitivity. To prioritize, each participant received five stickers to distribute across one or more identified problems. This visual voting method helped clarify collective priorities. The facilitator monitored whether participants understood the instruction and ensured the voting process was fair.

#### Ideate

In the Ideate session, we focused on determining the design of the tool. The process facilitator led the session, encouraging participants to freely contribute their ideas without considering obstacles.

#### Prototype and Test

The first and second authors developed a prototype based on the DT’s specifications, gathering additional feedback through individual discussions with members of the DT. In her objective role, the process facilitator ensured that all input was incorporated into the prototype. When substantive decisions needed to be made that did not directly come from the DT, these were always reviewed with the DT in the following session. A complete prototype was presented in March 2022, along with a feedback form for further input. After receiving feedback, the prototype was revised before proceeding to the test phase.

### Data Analyses

Each session generated a variety of qualitative input, including field notes taken by the researchers and process facilitator, written contributions from DT members, and audio recordings used as supplementary material. Following each session, the two researchers and process facilitator collaboratively reviewed all collected data to identify key insights ideas, and challenges expressed by the DT. These insights were then clustered into themes relevant that were considered relevant to become part of the tool. To ensure completeness and validity, interpretations by the researchers were brought back to the DT during subsequent sessions. This iterative and participatory process allowed us to collectively shape the prototype and informed the next steps within the Design Thinking process.

### Ethical Considerations

The first author explained Design Thinking and its role in the DT during the first meeting. Participants were asked verbal consent to (a) participate in the DT; (b) contribute to the tool development and a related scientific article; and (c) audio record the meetings. All participants agreed. This study was reviewed by the Research Ethics Review Committee (RERC) of the Faculty of Social Sciences (FSW) at Vrije Universiteit Amsterdam. The committee confirmed that further ethical review by the RERC was not necessary according to their guidelines, and therefore, the study was exempt from a full ethics review.

### Reflection on Design Thinking

The reflection on Design Thinking took place after the Design Thinking process had been completed. A reflection was carried out for each phase of the Design Thinking process with all authors of this article. The reflection focused on the following questions: (a) What were the outcomes of each individual phase, as well as the overall Design Thinking process? (b) What challenges did we encounter in each phase? (c) What information or insights, if known in advance, would have helped us better to navigate these challenges?

## Results

**Figure 1.**
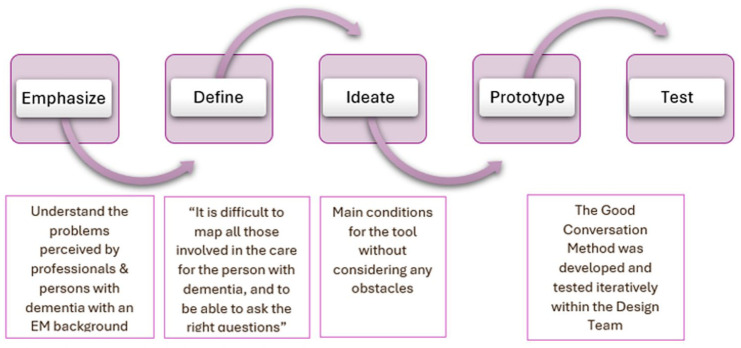
The Five Phases of the Design Thinking Process

First, we explain the process-related outcomes of Design Thinking. Second, we focus on the practical outcomes for nurses. The DT went through all five phases of Design Thinking (see Figure 1), resulting in the development of the Good Conversation Method.

### Design Thinking Process

#### Emphasize

In the Emphasize session, the DT reviewed insights from prior research presented by the first author. This phase combined scientific findings with personal experiences. The process facilitator monitored the conversations, ensuring the discussions stayed on track and intervening when necessary by posing questions to help the participants clarify their statements. For example, one conversation focused on communication issues faced by nurses when interacting with PwD with an EM background. The facilitator first listened to their discussion and then asked: “*What do these communication problems look like for you? Is it related to a language barrier?*” The professionals responded: “*It is not just the language barrier, but also maintaining contact and keeping appointments*.” The facilitator then emphasized: “*It is important to articulate the obstacles as concretely as possible, in this case, specifying what the communication issues look like*.” The DT added their own experiences to the table of encountered obstacles (Table 1), presented *in italics.* The results presented from the literature were recognizable to the participants of the DT, and they had their own examples which reinforced the findings from literature.

#### Define

The voting process resulted in two obstacles receiving the highest number of votes. The process facilitator decided it was important to include the voice of all DT members and suggested defining one issue combining the two selected most urgent obstacles.

#### Ideate

At the start, it was difficult for the participants to think freely and come up with ideas for the design of the tool. Questions from the process facilitator were necessary to assist the participants. Examples of responses included: “*The tool should help the professional understand the family situation and the wishes of the family*” (informal caregiver). The informal caregiver also formulated “*The tool should support the professional in facilitating the conversation, not only with the informal caregiver but with the entire family*.” Nurses stated: “*The tool should help the professional ask about the family structure*.” One of the students suggested that the tool should provide questions to ask family members.

#### Prototype and Test

After gathering input from the DT, the researchers collaborated with the process facilitator to develop an initial prototype. The prototype was then presented and tested within the DT, followed by the collection of further feedback to refine it. After several iterations between the prototype and testing phase with the DT, this process led to the final tool: the Good Conversation Method.

### The Good Conversation Method

The first phase of Design Thinking led to the following problem statement (see Figure 1): “It is difficult to map all those involved in the care of the PwD, and to be able to ask the right questions.” Table 1 provides an overview of the themes that were not included in the tool. As shown in Figure 1, the DT chose to develop a tool for nurses that supports them in (a) mapping the (in)formal network surrounding the PwD and (b) asking relevant questions to understand the cultural and religious identity of the PwD. The tool addresses key challenges in delivering culturally congruent care by supporting nurses in understanding the cultural identities of the PwD and improving culturally competent health care practices.

#### Mapping the (In)Formal Network

From the problem statement and the conversations within the DT during the first and second phases of Design Thinking, it became clear that the DT values mapping the (in)formal network. The DT indicated that there are sometimes (too) many people involved in the care for the PwD, making it difficult to maintain an overview. A member of the DT stated: “*Family relationships are important and influential in the caregiving process. Who is the decision-maker, and how do things typically work within the family?*” The lack of clarity results sometimes in the wrong people being addressed for continuation in health care. The DT identified the following barrier: “*The difference between the decision-maker and the informal caregiver that the nurses is in contact with, is not always clear*.” The DT emphasized that it is crucial to clearly identify the decision-maker, as this person often decides on important matters such as involving formal care. Therefore, the Ecogram (Hartman, 1978), an existing methodology, was chosen for this tool to support nurses in mapping the (in)formal network of the PwD. The DT decided to enhance the Ecogram with various options for representing relationships (tasks, positive and negative interactions with each other and with the PwD), making it clearer for the professional to understand the nature of the relationships and to identify any problematic ones.

#### Asking Questions

A second important topic the DT formulated was being able to ask the right questions to the EM people without a sense of offending the other person or bringing up culturally sensitive topics in an inappropriate manner. The tool should be helpful in communicating with the PwD and their informal caregiver, and it should support in the conversation nurses have with them. The DT identified several barriers that nurses may experience in lacking the ability to ask questions. One barrier, for example, was: “*Nurses feel insecure about their skills, e.g., being able to ask the right questions*.” According to the DT, this insecurity was primarily observed among nurses without an EM background. In addition, the DT identified another barrier: “*Nurses have stereotypical beliefs and assumptions about EM people*.” Therefore, the DT decided to expand the Ecogram with conversation cards. These cards support the nurses in mapping the (in)formal network by asking questions and assessing the influence of culture and religion on their conversations. The conversation cards must have example questions the nurses could ask and should serve as an inspiration to motivate nurses to engage in open conversations. It became apparent that some members of the DT had much, and some had little experience in working with EM people. Therefore, they decided to make the conversation cards optional. Eight conversation cards were developed (see Table 2).

**Table 2.** An Overview of the Conversation Cards Within the Good Conversation Method.

Theme conversation card	Aim conversation card
(1) Cultural and religious identity	To explore the cultural and religious identity of the care recipient
(2) Role of the conversation partner	To identify the role of the conversation partner in case the conversation partner is not the care recipient (but maybe the informal caregiver or translator)
(3) Physical care	To identify the (unmet) needs regarding physical care of the care recipient
(4) Emotional care	To identify the (unmet) needs regarding emotional care of the care recipient
(5) Important decisions	To understand how important decisions are being made and identify the involved persons in those important decisions
(6) Professional care	To identify the (unmet) needs regarding professional care of the care recipient
(7) Other forms of care	To explore other forms of care that are involved but not discussed yet
(8) Reflection on the conversation	To reflect together with the care recipient on the conversation and the mapped (in)formal network

#### Overarching Theme: Cultural and Religious Identity

The DT identified the following barrier: “Informal caregivers feel a lack of person-centered health care” and unanimously agreed in the first prototype phase that the cultural and religious identity of the PwD should have a central place in the tool. Nurses should be supported in exploring cultural and religious identity. Therefore, the DT decided to include a conversation card with “Cultural and religious identity” as theme with questions to inspire the nurses to have a conversation about this theme. In addition to the separate conversation card for cultural and religious identity, the DT has chosen to address this theme in other cards also. For example, the card “Physical care” includes questions related to culture and religion: “Are there cultural aspects that need to be considered in physical care?.” A question on the “Professional care” card regarding culture and religion is as follows: “Do nurses understand the cultural or religious background? If so, how do you notice that? If not, what needs to be done for them to understand?.” The final version of the Good Conversation Method was tested within the DT. All participants agreed that this final version can be shared and can be used to support nurses in strengthening their cultural competence.

### The Good Conversation Method Explained

The Good Conversation Method is a practical tool that health care professionals can use to map the (in)formal network of a PwD, using a genogram. In addition to identifying members of the network it also allows professionals to represent the nature of the relationship (strong, weak or absent) and the roles of each involved person. The method includes a set of conversation cards with guiding questions designed to explore the cultural and religious identity in a structured way.

## Discussion

The objective of our study was to develop a tool that focuses on strengthening cultural competence and to reflect on the Design Thinking process. We developed the tool in co-creation with end users and stakeholders, applying the Design Thinking methodology. The DT determined that the tool should support nurses with the overall aim in mapping the (in)formal network and asking questions related to the cultural and religious identity of the person with an EM background and their families.

### Good Conversation Method

The DT selected two main topics for the tool and connected two tools to them: the ecogram (Hartman, 1978), enhanced by conversation cards with questions to support the nurses. This decision stemmed from a prioritization process, leaving out topics like “lack of knowledge about dementia within families” and “lack of awareness about dementia care professionals,” which are also recognized as barriers for EM people in accessing health care (Berdai Chaouni & De Donder, 2019; Hossain & Khan, 2019; Mountford & Dening, 2019; Mukadam et al., 2011; Parveen et al., 2018). Yet, with the need to prioritize, the focus was more on the perceived barriers of the health care professional, as the uncertainty which questions to ask, than on the barriers as perceived by the PwD (see Table 1). Thus, this Design Thinking process prioritized the attitudes and uncertainties of nurses over other aspects. Previous research emphasizes that the challenge goes beyond asking questions (Duran-Kiraç et al., 2023). During the sessions of the DT, it was realized that the real challenge involves the attitudes of nurses toward EM people. Studies reveal that professionals often view EM people as “the other” with different views and needs than one’s own, causing insecurity and a sense of incompetence (Claeys et al., 2023). Furthermore, asking appropriate questions and maintaining an open attitude are crucial for strengthening cultural competence (Duran-Kiraç et al., 2023). Based on the DT discussions, the Good Conversation Method may support professionals in fostering openness, asking questions, and engaging meaningfully with “the other.”

The DT emphasized the importance of acknowledging the cultural and religious identity of PwD. Previous studies show that both PwD and their informal caregivers often feel that nurses overlook their cultural and religious needs (Duran-Kiraç et al., 2022). Other research has shown that health care services fail to meet cultural needs of EM people, with a lack of intercultural person-centered care (Chaouni et al., 2020; Nielsen et al., 2020; Sagbakken et al., 2018). Duran-Kiraç et al. (2022) recommend educating nurses to address the cultural, religious, and linguistic needs of patients. Both nurses and informal caregiver within the DT acknowledged the empirical findings of the project and the literature on this matter. Nurses stated that they encounter challenges and find it difficult to clarify cultural and religious needs.

### Design Thinking: Lessons Learned

#### The Composition of the DT

The DT played a key role in addressing the issue of strengthening cultural competence among nurses through co-design. Co-design encourages active involvement from users and stakeholders in developing new solutions or services (Lam & Pitsaki, 2018). The composition of the DT is crucial in co-design, as its members decide on the challenge to tackle and the type of tool needed. They should be familiar with the challenge it aims to tackle. Both the compostion of the team and their interactions determine how Design Thinking is implemented. It is therefore important to ensure equal participation patterns within the DT. Unequal participation patterns between members of the DT can lead to tensions (Goldman et al., 2014).

##### The first lesson from our Design Thinking process is the importance of the participation of all stakeholders in the Design Thinking process

One study found that conflicts within teams can stem from a lack of leadership of task distribution issues (Aflatoony et al., 2018). To ensure equal participation and clear task distribution, an independent process facilitator was appointed to ensure all members had an equal voice.

##### The second lesson from our Design Thinking process is the importance of appointing a process facilitator, whose main tasks include ensuring equal participation from all members of the DT and providing space for participating researchers (who also can be stakeholders)

In Design Thinking particularly, it is important to engage all stakeholders, including researchers. This makes it even more important in this context that the involved researchers can delegate process related tasks to the process facilitator.

#### Aligning Team Differences

Each involved stakeholder within the DT has a different frame of reference. For example, nurses view the problem through the lens of their practical work, while the informal caregiver had her own experiences with dementia care. To align everyone’s knowledge and insights, we provided the DT with information from previous research (Duran-Kiraç et al., 2022; Duran-Kiraç et al., 2023). We believed this would help them approach the problem from a shared perspective. Although our effort resulted in an overview of existing knowledge and insights, we wonder if this was enough to reach agreement on what is truly important within the DT. We did not receive any signals of conflicting viewpoints within the DT. However, we should still take a moment to reflect on this by checking it with the DT. The Design Thinking process does not provide guidance for researchers or process facilitators on how to navigate potential conflicts (Verganti et al., 2021).

##### The third lesson learned from our Design Thinking process is that researchers and process facilitators need to prepare on how to navigate through and work with possible conflicting viewpoints within the DT

Literature identifies three types of conflict: cognitive, relationship, and process conflicts. Relationship conflicts involve emotional differences and poor relationships, while process conflicts relate to disagreements about timing, planning and scheduling. Cognitive conflicts occur when persons have different beliefs or perspectives (Ying & Lei, 2020). Research shows that relationship and process conflicts negatively affect productivity (Ying & Lei, 2020), but cognitive conflicts can lead to fruitful discussions and more ideas (Badke-Schaub et al., 2007), which are essential in Design Thinking. While not yet a formal part of Design Thinking, methods like Empathy Mapping can help. An Empathy Map is a collaborative tool that visualizes what we know about a specific user, helping to create a shared understanding of their needs and support decision-making (Sinansari et al., 2023).

##### The fourth lesson learned from our Design Thinking process is that researchers should always be mindful of the various viewpoints of stakeholders, even when no conflict seems to be present at first

An Empathy Map can be helpful to ensure that no conflicting viewpoints are present that could slow down or hinder the Design Thinking process.

#### Define

We chose the most urgent problem by voting on issues identified by the DT. However, the voice of the PwD was missing. An opportunity of Design Thinking is that all stakeholders are involved in the DT. In the context of our objective, this also should include PwD. Meetings were held during COVID-19. Although restrictions were eased, informal caregivers felt it was too risky for PwD to participate. While understandable, we could have explored alternative ways to include PwD. Meaningful partnerships in scientific research include inclusion, representation and shared decision-making (Walter et al., 2025). It is possible that the inclusion of PwD could have influenced the decision-making process regarding the issues identified by the DT, potentially leading to a different problem being chosen to develop a tool for.

##### Lesson five is the need to consider how to include all perspectives of the stakeholders, including of PwD in the Design Thinking process

*Researchers should be prepared to think about participation of stakeholders in alternative ways*. In cases where PwD may find it challenging to participate, researchers should explore other possibilities to gather input. Previous studies have already demonstrated that there are various methods to facilitate their involvement (Collins et al., 2024).

#### Prototype and Test

We chose to conduct the Testing phase within the DT. This meant participants tested the tool after each prototype with other DT members. A key lesson for us is that the testing phase may need to be more extensive. By expanding the test to the professionals’ practice, you can gather more extensive input on the tool’s effectiveness within the process of Design Thinking. However, the methodology requires flexibility but the researcher has a time frame to deal with. Design Thinking requires to go back and forward between the different phases until a good prototype is developed and tested (Roddy & Polfuss, 2020). This iterative process can sometimes take longer than the time available to the researcher (due to budget constraints, for example). There is no maximum number of times the phases can be revisited within Design Thinking, but the research output requires framing of the project to achieve valid and transferable findings (Jacobs, 2017).

##### The final lesson learned from our Design Thinking process is that researchers should consider the potential conflict between the reality of limited time and the Design Thinking process’s need for multiple iterative cycles across different phases

While time management is crucial in any research, it is especially important in Design Thinking because the process is inherently iterative and requires continuous updates based on feedback. We advise researchers to allow ample time for iteration between phases, including in the planning to prevent iterations from being rushed through too quickly.

#### Implications

The Good Conversation Method offers nurses a practical tool to engage in more culturally sensitive communication with EM people. This supports the delivery of culturally competent health care. In addition, the study also provides valuable insights into the Design Thinking process that may inform future research. Lessons learned can contribute to the further refinement of Design Thinking as a method for co-creation to contribute to more culturally responsive health care innovations.

#### Limitations

A limitation of this study is the absence of direct input from PwD during the DT sessions, particularly given the study’s focus on person-centered care. We acknowledge that incorporating perspectives of PwD is essential to understand their needs and preferences. Alternative approaches are needed to ensure their voices are included, such as conducting individual interviews outside of the group sessions, or using proxy interviews with informal caregivers. These strategies could help capture the experiences of PwD more effectively and enhance inclusivity.

#### Future Directions

While the Good Conversation Method was iteratively developed and refined within the DT, it has not yet been evaluated in clinical practice. To evaluate its potential to strengthen nurses’ cultural competence, future research should focus on piloting the method in health care settings. A longer-term implementation is needed in which nurses apply the tool repeatedly in their daily work with EM PwD. We recommend conducting a pilot study in which a diverse group of nurses (such as dementia case managers and practice nurses) integrate the GCM into their interactions. This allows a comprehensive evaluation of the effectiveness in fostering an open attitude, encouraging culturally sensitive questioning, and supporting the identification and understanding of the cultural and religious identity of PwD with an EM background. Such a pilot should also gather in-depth feedback from health care professionals on its usability. These insights are crucial in assessing how, when, and under what conditions the GCM contributes to culturally competent health care, and identifying opportunities for further implementation.

## Conclusion

Design Thinking has contributed to involving stakeholders in the development of the Good Conversation Method that aims to strengthen nurses’ cultural competence. We have learned important lessons that can improve the Design Thinking process. First, it is important to have all stakeholders participated in the Design Thinking process. Second, appointing a dedicated process facilitator proved crucial, enabling researchers to concentrate on content. Third, researchers must be prepared to navigate and manage conflicting viewpoints within the DT. Fourth, it is important to be mindful of the various viewpoints of stakeholders, even when no conflict seems to be present at first. Fifth, it is important to find ways to include all stakeholders, and sixth, researchers should consider the potential conflict between limited time and the Design Thinking process’s need for multiple iterative cycles.

## Supplemental Material

sj-pdf-1-tcn-10.1177_10436596251370604 – Supplemental material for Using Design Thinking to Develop a Tool for Strengthening Nurses’ Cultural Competence: Lessons Learned Supplemental material, sj-pdf-1-tcn-10.1177_10436596251370604 for Using Design Thinking to Develop a Tool for Strengthening Nurses’ Cultural Competence: Lessons Learned by G. Duran-Kiraç, R. Uittenbroek, H. van Hout and M. I. Broese van Groenou in Journal of Transcultural Nursing
